# Supplementary material for: New Characterization of Multi-Drug Resistance of Streptococcus suis and Biofilm Formation from Swine in Heilongjiang Province of China
Source: Antibiotics (Basel). 2023 Jan 10;12(1):132. doi: 10.3390/antibiotics12010132 (PMC9854593; doi:10.3390/antibiotics12010132)
Supplement: Supplementary file 1 [file antibiotics-12-00132-s001.zip › antibiotics-2117646-supplementary.pdf]

Supplementary Material

New characterization of Multi-drug Resistance of *Streptococcus suis* and Biofilm Formation from swine in Heilongjiang Province of China

Chun-Liu Dong[1,2†], Rui-Xiang Che,[1,3†], Tong Wu[1], Qian-Wei Qu[1], Mo Chen[1]， Si-Di Zheng[1], Xue-Hui Cai[4], Gang Wang[4,5*],Yan-Hua Li[1, 2*]

1. *College of Veterinary Medicine, Northeast Agricultural University, Harbin, Heilongjiang 150030*
2. *Heilongjiang Key Laboratory for Animal Disease Control and Pharmaceutical Development, Harbin, China*
3. *College of Animal Science and Veterinary Medicine, Heilongjiang Bayi Agricultural University, Daqing, China*
4. *State Key Laboratory of Veterinary Biotechnology, Harbin Veterinary Research Institute, Chinese Academy of Agricultural Sciences, Harbin, China*
5. *Department of Basic Veterinary Medicine, College of Veterinary Medicine, Shandong Agricultural University, Taian, China*

*Correspondence to: Professor Yanhua Li, E-mail: liyanhua@neau.edu.cn (Y.-H.Li),
and Gang Wang, wanggang@caas.cn (G. Wang)
† **Co-first author**: These authors have contributed equally to this work.

Abbreviations:

Table S1. **The different serotypes of *Streptococcus suis.***

Serotype	Number	Region
2	1	Harbin-2
2	9	Harbin-1; Qiqihar
4	15	Harbin-1; Da Qing；Harbin-3
9	1	Harbin-2
no	1	Harbin-2
no	1	Da Qing
no	1	Suihua

Table S2. MIC distribution (%) for *Streptococcus suis* isolates from June 2017 to August 2018.

Antimicrobial agent		Number of strains with MIC (µg/mL)													MIC_{50}[a] (µg/mL)	MIC_{90}[b] (µg/mL)
		≤0.06	0.12	0.25	0.5	1	2	4	8	16	32	64	128	>128		
Macrolides	Erythromycin							1	1		2		2	23	>128	>128
	[c] Tylosin									1	2	2	4	20	>128	>128
Lincosamides	[c] Lincomycin											3	7	19	>128	>128
Aminoglycosides	[c] Kanamycin							1		1		1	4	22	>128	>128
	Gentamicin					1		2	2	1	2	6	3	12	128	>128
Tetracyclines	[c] Oxytetracycline	1		1		2	1			8	8	6	1	1	32	64
	Tetracycline					1		1	2	10	6	4		5	32	>128
	[c] Chlortetracycline	1				1		4	6	8	7	2			16	32
Chloramphenicol	Chloramphenicol	1						2		7	1	4	1	13	64	>128

	Florfenicol	1					3				5	4	2	14	128	>128
	Ceftiofur	1			1		2	1	1	8	3	7	3	2	32	>128
β-lactams	[c] Cefquinome	1			1	2	1	2	1	4	9	4	3	1	32	128
	[c] Amoxicillin		2	1		3	2	1	1	1	3	6	6	3	64	>128
	Penicillin K	1	2			1		2		1	2	5	8	7	128	>128
	[c] Ofloxacin	1	1			2	8	6		4	6			1	4	32
Fluoroquinolones	[c] Ciprofloxacin	1	2		2	2	1	7	2	4	5			3	4	>128
	Enrofloxacin	1	2			6	3	2	1	8	3	1	2		8	64
	[c] Dafloxacin	1	1	1	1	2	5	4	4	2	7		1		4	32

a,b MIC 50 and MIC 90 are the lowest MIC at which at least 50% and 90% of the isolates in the test population were inhibited.
c For Tylosin, Lincomycin, Kanamycin, Oxytetracycline, Chlortetracycline, Cefquinome, Amoxicillin, Ofloxacin, Ciprofloxacin and Dafloxacin no clinical breakpoint is available

Table S3 Isolation and Identification of *Streptococcus suis.*

Isolate	ST (*cps*)	Site of Isolation	Isolation of City	Year	Antimicrobial susceptibility profiles	AMR genes	Medicine history
AHD-110-6-2	4	nasal	Harbin-1	2018	ERY,TET	*lnu(B), mefE*	This did not receive any recent antibiotic therapy.
AY18-2	4	nasal		2018	ENR, CHL, ERY, PK, TET, FFC, CEF	*ermB, lnu(B), mefE*	
AH94-4	4	nasal		2018	TET, ERY, GEN, ENR	*ermA, ermB, aph(3')-IIIa, lnu(B), ermTR, mefE, tetO*	
AZ45-1	2	nasal		2018	ENR, CHL, ERY, PK, TET, FFC, CEF, GEN	*aph(3')-IIIa, ermA, ermTR, mefE,msrD, tetM*	
AZ52-1	2	nasal		2018	ENR, CHL, ERY, PK, TET, FFC, CEF, GEN	*ermA, ermB, aph(3')-IIIa, ermTR, mefE, tetO*	
AH94-2	4	nasal		2018	ENR, CHL, ERY, PK, TET, FFC, CEF, GEN	*ermB, aph(3')-IIIa, lnu(B), tetO, tetM, mefE*	
YF74-2	no	nasal	Harbin-2	2018	ENR, ERY, PK, TET, CEF, GEN	*ermA, aph(3')-IIIa, ermTR, mefE, tetM*	This did not receive any recent antibiotic therapy.
YHB13-2	2	nasal		2018	GEN, ERY, ENR	*ermB, aph(3')-IIIa, lnu(B), mefE*	
YB58-2	9	nasal		2018	ENR, CHL, ERY, PK, TET, FFC, CEF, GEN	*ermB, aph(3')-IIIa, lnu(B), mefE, tetM*	
H1-2	4	nasal	Harbin-3	2018	ENR, CHL, ERY, PK, TET, FFC, CEF, GEN	*ermA, ermB, aph(3')-IIIa, ermTR, msrD, mefE,tetM*	This farm only used tylosin to treat the pigs.
GJ1-2	4	joint		2018	ENR, CHL, ERY, PK, TET, FFC, CEF, GEN	*ermA,ermB, lnu(B), tetO, ermTR, mefE*	
HB10-1	4	nasal		2018	ENR, CHL, ERY, PK, TET, FFC, CEF, GEN	*ermA,ermB, aph(3')-IIIa, tetM, ermTR, mefE*	
B9-1	4	nasal		2018	CHL, ERY, TET, FFC, CEF, GEN	*ermB, tetO*	
H3-1	4	nasal		2018	ENR, CHL, ERY, PK, TET, FFC, CEF, GEN	*ermA,ermB, aph(3')-IIIa, lnu(B),ermTR, tetM*	
Y10-2	4	nasal		2018	ENR, CHL, ERY, PK, TET, FFC, CEF, GEN	*ermB, aph(3')-IIIa, mefE*	

S-1	4	abdomen		2018	ENR, CHL, ERY, PK, TET, FFC, CEF, GEN	*ermB*, *aph(3')-IIIa*, *lnu(B)*, *ermA*, *tetO*, *ermTR*, *mefE*	
S-2	4	abdomen		2018	ENR, CHL, ERY, PK, TET, FFC, GEN	*ermA*,*ermB*, *aph(3')-IIIa*, *lnu(B)*,*ermTR*, *mefE*, *tetO*	
B1-2	4	nasal		2018	ENR, CHL, ERY, PK, TET, FFC, CEF, GEN	*ermB*, *aph(3')-IIIa*, *ermTR*, *mefE*, *tetM*	
2-5	no	lung	Suihua	2017	ENR, CHL, ERY, PK, TET, FFC, CEF, GEN	*aph(3')-IIIa*, *tetM*	This did not receive any recent antibiotic therapy
ZL695-2	no	nasal		2018	CHL, ERY, PK, TET, FFC, CEF, GEN	*ermA*,*ermB*, *aph(3')-IIIa*, *mefA*, *ermTR*, *mefE*	
DZ001-2	4	nasal	Da Qing	2018	CHL, ERY, PK, TET, FFC, GEN	*ermB*, *aph(3')-IIIa*, *msrD*	This farm used tylosin to treat the pigs.
DY12-2	4	nasal		2018	CHL, ERY, PK, TET, FFC, CEF, GEN	*ermA*, *aph(3')-IIIa*, *ermTR*, *mefE*,*tetO*	
LQ-5	2	nasal		2017	ENR, CHL, ERY, PK, TET, FFC, CEF, GEN	*ermB*, *aph(3')-IIIa*，*tet M*, *lnu(B)*	
D-1	2	nasal		2017	ENR, CHL, ERY, PK, TET, FFC, CEF	*ermA*,*ermB*, *tetM*, *lnu(B)*, *ermTR*, *mefE*	
G-3	2	nasal		2017	ENR, CHL, ERY, PK, TET, FFC, CEF	*ermA*,*ermB*, *aph(3')-IIIa*, *lnu(B)*, *ermTR*, *mefE*	
LL-3	2	nasal	Qiqihar	2017	CHL, ERY, PK, TET, FFC, CEF	*ermB*, *tetM*, *lnu(B)*	This farm used tylosin, lincomycin , gentamicin, sulfonamide to treat the infected pigs.
LL-1	2	nasal		2017	CHL, ERY, PK, TET, FFC, CEF, GEN	*ermA*,*ermB*, *ermTR*	
K-5	2	nasal		2017	ENR, CHL, ERY, PK, TET, FFC, CEF, GEN	*erm A*, *ermB*, *aph(3')-IIIa*, *tetO*, *lnu(B)*, *ermTR*,*mefE*	
Z-5	2	nasal		2017	CHL, ERY, PK, TET, FFC, CEF, GEN	*aph(3')-IIIa*, *tetM*, *lnu(B)*	

Table S4 Primers used for PCR analysis.

Primer	Sequence（5’-3’）
Gdh-S	GCAGCGTATTCTGTCAAACG
Gdh-R	CCATGGACAGATAAAGATGG
SS2-S	TTCGTATTAACTTACTTGGCGT
SS2-R	TAAATCCCCATATGCCAAATCC
SS4-S	TGATATTGGCTATCTTTTGGGG
SS4-R	TTCCCCCTTCAAATAAACTCTG
SS7-S	AAAATTCGTTCCATTGTAGGTG
SS7-R	TGAAGTTGAAGCTGGTGATAAA
SS9-S	TGAAAGTAGGTATATCTCAGCA
SS9-R	AAAGAATTGAATCCCACCTGAG

Table S5 Primers used for drug resistance gene PCR analysis.

Primer	Sequence（5’-3’）
erm(A)-S	TCTAAAAAGCATGTAAAAGAA
erm(A)-R	CTTCGATAGTTTATTAATATTAGT
erm(B)-S	GAAAAGGTACTCAACCAAATA
erm(B)-R	AGTAACGGTACTTAAATTGTTTAC
mef A-S	AGTATCATTAATCACTAGTGC
mef A-R	TTCTTCTGGTACTAAAAGTGG
mef E-S	CGTAGCATTGGAACAGC
mef E-R	TCGAAGCCCCCTAATCTT
msrD-S	CCTTATCGGCACAGGTTCAT
msrD-R	GCCTTCCGGAGCTCCTACTT
ermTR-S	TTGGGTCAGGAAAAGGA
ermTR-R	GGGTGAAAATATGCTCG
tet(O)-S	TGCGGCAAGGTATTCTTAAAT
tet(O)-R	ATTTTATATGACTTTTGCAAGCTG
tet(M)-S	GTGGACAAAGGTACAACGAG
tet(M)-R	CGGTAAAGTTCGTCACACAC
lnu(B)-S	CGTGGGGAATTTCATTTCCTTTC
lnu(B)-R	CGTTGATTCCCATCAACCATAG
aph (3’)-IIIa -S	GGCTAAAATGAGAATATCACCGG
aph (3’)-IIIa -R	CTTTAAAAAATCATACAGCTCGCG
